# *Chaenomeles* Species—Characteristics of Plant, Fruit and Processed Products: A Review

**DOI:** 10.3390/plants11223036

**Published:** 2022-11-10

**Authors:** Natalia Marat, Marzena Danowska-Oziewicz, Agnieszka Narwojsz

**Affiliations:** Department of Human Nutrition, Faculty of Food Science, University of Warmia and Mazury in Olsztyn, 10-718 Olsztyn, Poland

**Keywords:** *Chaenomeles*, fruit, processed products, bioactive compounds

## Abstract

This literature review deals with the characteristics of *Chaenomeles* species and the physicochemical properties of *Chaenomeles* fruits. These fruits belong to a group with a low content of monosaccharides and a favorable ratio of fructose to glucose. They exhibit a low pH value and sour taste; therefore, they are not eaten in a raw form. They have a high concentration of bioactive compounds, such as polyphenols, vitamin C, organic acids, dietary fiber and pectins. The physicochemical properties of processed *Chaenomeles* fruits, i.e., freeze-dried, juices, syrups, candied fruit, jam, powder and chips, are presented in the manuscript. Also mentioned are the seeds and their use in the production of oil and seed gum. Of the products described in the paper, seed oil deserves greater attention, as it is characterized by a high content of unsaturated fatty acids, mainly oleic and linoleic, and low susceptibility to oxidation.

## 1. Introduction

Consumers are increasingly interested in the health benefits provided by food components. Fruit and vegetables deserve special attention as they are a valuable source of bioactive compounds that determine the proper functioning of the human organism [1,2]. A good source of bioactive compounds is Japanese quince (*Chaenomeles japonica*) fruit. The Japanese quince shrubs originate from Asia, but currently, they are cultivated mainly in Europe, within the Baltic Sea basin [3,4,5]. Japanese quince fruit, due to its high concentration of bioactive compounds and their effects on the human body, is used in the food, pharmaceutical and cosmetic industries [6]. Because of the fruit’s hardness, astringency and acidity, they are not intended for direct consumption but are a good material for processing [7,8]. Interest in the flavour and aroma of the Japanese quince has resulted in its introduction for consumption in the form of jams, jellies, syrups, juices, additions to ice-cream and aromatic wines and liquors [9].

Traditional ethnomedicine is on the rise as plants were for ages known and used for disease treatment purposes. According to traditional Chinese medicine, fruits that have a sour taste and are naturally warm have the ability to calm the liver, harmonise the stomach, relax tendons and muscles and eliminate moisture, which can prevent or treat intestinal inflammation, rheumatism, dysentery, cholera, beriberi, sunstrokes, depression and vitamin C deficiency syndrome [10,11]. Saifulazmi et al. [12] reviewed the literature and reported that *Chaenomeles sinensis* is polyphenol-rich fruit used in traditional Chinese medicine for treating throat diseases and which presently has potential in preventing influenza virus infections. Li et al. [13] mentioned the use of *Chaenomeles* fruits in Korean and Chinese medicine also as treatment for anaphylaxis and neurodegenerative diseases, as well as their antihyperuricemic, antiacetylcholinesterase and antidiabetic properties. Thanks to its properties, the *Chaenomeles speciosa* fruit has long helped to control illnesses such as sunstroke, migraine and enteritis [10]. Glucosides contained in *C. speciosa* exhibit immunoregulatory and anti-inflammatory activity [14]. *C. speciosa* has been proven to be effective in dopamine transport and to protect against Parkinson’s disease [10]. A study conducted with the use of mice showed the ability of this fruit to inhibit the development of neoplastic cells and to enhance the immune system of the organism [15]. *C. speciosa* fruit extracts exhibit potent anticancer, antioxidant, antiviral, antimicrobial, antihyperlipidemic and antihyperglycemic properties due to their content of various phytochemicals [16]. The results of Owczarek et al. [17] indicate that a flavanol preparation from *C. japanese* fruit reduces the viability of colon cancer cells. Itoh et al. [18] reported that phenolic compounds from *C. sinensis* sarcocarp have the potential to protect against skin aging through anti-collagenase activity. The study by Sancheti et al. [19] revealed the protective effect of ethyl acetate fraction of *C. sinensis* fruit against a diabetic dementia model in laboratory animals.

Numerous studies have demonstrated the health-promoting properties of extracts from the Japanese quince fruit [4,14,20]. It is a raw material rich in bioactive compounds, mainly phenolic compounds, vitamin C and pectins [3]. Phenolic compounds play an important role in the human organism and exhibit a number of physiological properties. They exhibit anti-inflammatory, anti-allergic, anticoagulant, antibacterial, vasodilatory and antioxidant properties [21]. Moreover, they prevent diabetes mellitus, neoplasms and obesity [22]. Vitamin C regulates arterial blood pressure, enables the proper course of the oxidative degradation of tyrosine, accelerates wound healing, lowers blood glucose levels, affects the absorption of calcium and non-haem iron, is involved in the synthesis of transmitters and hormones, reduces oxidative stress caused by physical effort and participates in fat and steroid metabolism [23,24,25]. Pectins contained in fruit and vegetables have a beneficial effect on the gastrointestinal microflora and the metabolism of lipids, cholesterol and glucose; reduce the activity of toxins released by *Escherichia coli*; and protect against *Streptococcus pneumoniae* infections [26,27,28,29]. Moreover, they exhibit anti-inflammatory activity and are used in the prevention and treatment of gastric ulcers [30,31].

Plant bioactive compounds are presently considered safe and economically advantageous components which constitute a base for around 30% of the pharmaceutical market [12,19]. The research for new, and potentially precious from a pharmaceutical viewpoint, bioactive components in *Chaenomeles* fruits is in progress [13]. In the future, the fruit of *C. japonica* may become a component of functional foods or dietary supplements, thanks to its high content of pectin and fibre [32].

The aim of this study was to systematise the existing knowledge on both the characteristics of *Chaenomeles species* and the physicochemical properties of the fruit and products made from them.

## 2. *Chaenomeles* as a Crop Plant

The Latin name *Chaenomeles* is derived from the Greek word “*chaino*”, which means “to gape”, and “*melon*”, which means “an apple” [5]. *Chaenomeles* is a plant belonging to the *Rosaceae* family and the *Pomoideae* subfamily [5,7,14,33]. At present, four East Asian species of this plant are widely known: *Chaenomeles thibetica* Yu, *Chaenomeles cathayensis* Schneider, *Chaenomeles speciosa* Nakai and *Chaenomeles japonica* Lindl. In China, *Chaenomeles sinensis* Koehne is additionally distinguished. There are also mixed species, e.g., *Chaenomeles californica* (*C. cathayensis x C. japonica*) and *Chaenomeles superba* (*C. speciosa x C. japonica*) [33,34,35]. The species *Chaenomeles speciosa* is naturally found in the eastern, central and south-western parts of China and is currently cultivated all over the world. In the eastern part of China, in the Anhui province, *C. speciosa* is considered a plant of great importance and is used for medicinal purposes [10,14,35].

The Japanese quince (*Chaenomeles japonica*) was introduced to Europe in 1869. Initially, it was considered an ornamental plant, although in certain countries it was appreciated as a fruit bush [36]. In the second half of the 20th century, interest in the fruit increased significantly due to the possibility of growing the plant in Europe, mainly in the Baltic Sea countries. In 1951, the cultivation of Japanese quince shrubs commenced in Latvia. In Poland, since 1978, the fruit has been investigated for its antioxidant properties and used in the food industry [3,7]. As a part of the cultivation programme, methods for the Japanese quince shrub’s cultivation and breeding in Lithuania, Latvia and Sweden were developed in 1992. In 1993, there was a breakthrough in Japanese quince cultivation. In Lithuania and Latvia, up to 400 ha of fruit shrub cropland was recorded [7,37]. Currently, Japanese quince shrubs are cultivated mainly in Lithuania, Estonia, Latvia, Sweden, Finland and Poland [3,4].

*C. japonica* is a dwarf plant reaching 0.6-1.2 m in height. In natural habitats, it grows on hillsides (100–2100 m ASL) and on lake and river banks in central and southern Japan. The leaves are ovate-shaped with serrated edges and a length of 3–5 cm. The species *C. thibetica*, *C. cathayensis* and *C. speciosa* are mainly found in China (Yunnan and Tibet), where they overgrow hillsides, ravines and forests. In comparison, *Chaenomeles thibetica* reaches a height of 1.5–3 m and grows at an altitude of 2700-3600 m ASL; *C. cathayensis* reaches a height of up to 6 m and grows at an altitude of 900–2500 m ASL; and the species *C. speciosa* reaches a height of 2–5 m and grows at an altitude of 200–1700 m ASL. The leaves of the above-mentioned species are oval-shaped and serrated, with a variable size of 3–9 × 1.5–5 cm [7,33,37].

The flower buds usually appear on the *Chaenomeles* shrub in summer and autumn. Sometimes, short shoots appear in spring and late autumn, while long shoots appear from late spring to late autumn (*C. speciosa*—April and May; *C. japonica*—the end of April and May). Each fruit shrub species forms unisexual (male or female) flowers. The sex of the flower can be distinguished by the size and shape of one of the parts forming the basal portion of the calyx (hypanthium). A short, bowl-shaped hypanthium indicates male flowers on the shrub [7,33,38]. Depending on the plant species, the colour of the petals can vary from white, through orange and pink, to dark red [7]. For example, the flowers of *C. speciosa* are bright red, and the colour of the flowers of *C. japonica* ranges from white to pink [33].

The *Chaenomeles* fruit is characterised by a varied size and an irregular shape. The species *C. japonica* has the smallest fruit of all shrubs of this genus. Its weight does not exceed 50 g, and the diameter is approx. 4 cm. Up to 80 seeds are formed in the seed cavity. The fruit is hard and astringent and contains low sugar levels. When fully ripe, it turns yellow [5,7]. The fruit of *C. thibetica* is characterised by an elongated shape resembling that of the pear fruit. It reaches a length of 6–11 cm, and its diameter is 5–9 cm. *C. cathayensis* has the largest fruit of all *Chaenomeles* species, with a length of 15 cm and a diameter of 8 cm. The fruit’s weight can reach 600 g, and there are up to 120 seeds in the seed cavity. The fruit of the *C. speciosa* species is 4–7 cm long, has a diameter of 3–6 cm and its weight does not exceed 140 g. Approximately 100 seeds can be found in the seed cavity [7,33].

*Chaenomeles* plants are also interesting from the agricultural point of view. Fedulova et al. [39] documented an occurrence of phytopathogens and phytophages on the leaves and fruits of *Chaenomeles*. In general, the authors reported a satisfactory condition of the investigated plants; however, the presence of particular pathogens and phytophages was observed. This was ascribed to the closeness with other plantings of the Rosaceae family in the plants collection under investigation. Nevertheless, the authors were able to point out disease-resistant cultivars of *Chaenomeles*. Similar observations related to *C. japonica* being a relatively healthy plant were noted earlier by Norin and Rumpunen [40], who also indicated fungi as a main potential threat for that crop, especially when grown in greater areas. Due to its low soil and climate requirements, resistance to pests and no need to fertilize, Japanese quince is recommended for cultivation, especially in organic farming [6].

## 3. Physicochemical Properties of *Chaenomeles* Fruit

The content of selected compounds in *Chaenomeles* sp. fruits is summarized in Table 1. The dry matter (DM) contents in the fruit of *Chaenomeles x superba* and *Chaenomeles* spp. are comprised of 17.00 mg/100 g of fresh matter (FM) and 15.51 mg/100 g FM, respectively [5]. The Japanese quince fruit is rich in organic acids, mainly malic accompanied by quinic and succinic acids. The former contributes to the high acidity and low pH value (2.4–2.8) of the fruit. The acidity of the fruit ranges from 2.6 to 5.6%, expressed as citric acid [34]. The Japanese quince fruit is poor in monosaccharides. The fructose content is 6.31 g/100 g DM, and the glucose content is 10.95 g/100 g DM [41]. The fruit is characterised by a high content of polysaccharides, e.g., cellulose, hemicellulose and pectins, which are components of dietary fibre. A hundred grams of the fruit’s dry matter contain 18 g of cellulose and 3 g of hemicellulose. The average pectin content is 11 g/100 g DM (1.4 g/100 g FM). These values are comparable with the pectins content in apples. The pectins are mainly located in the fruit’s flesh [38,42,43].

Phytochemical research results indicate the presence of secondary metabolites in individual parts of the Japanese quince plant. In the roots, the presence of daucosterol, ursolic acid, oleanolic acid, pomolic acid, prunasins and epicatechins was demonstrated. The leaves contain epicatechin and flavonol glycosides, while in the fruit, monoterpene glucosides, leucoanthocyanines, epicatechin and roseoside are present [45]. Seglina et al. [3] indicated that the fruits were rich in bioactive compounds, e.g., phenolic compounds, vitamin C, organic acids, aromatic compounds and pectins [3].

According to Du et al. [35], the fruit is characterised by a high level of total phenolic compounds. For example, *C. japonica* contains phenolic compounds at a level of 19.35 mg GAE /g FM, while *C. speciosa* and *C. thibetica* contain levels of 46.92 mg/g FM and 46.28 mg/g FM, respectively. Urbanavičiūtė et al. [46] analysed the content of phenols in new varieties of Japanese quince fruit and showed significant differences between the varieties “Darius”, “Rasa” and “Rondo”. The authors stated that the “Rasa" variety was characterized by the highest content of phenolic compounds (4366 mg GAE/100 g DM). A study using HPLC-DAD/ESI-MS/MS demonstrated that the Japanese quince fruit contained 24 phenolic compounds, of which 20 were flavan-3-ols such as catechin, epicatechin and procyanidins, which accounted for approx. 94–99% of all polyphenols. The other polyphenols included chlorogenic acid, a chlorogenic acid isomer, and two quercetin glycosides. Catechin was found to be present at a level of 2.9 mg/100 g FM, while procyanidin B1 was at a level of 9.8 mg/100 g FM. The fruit was also noted to contain the greatest amount of epicatechin (11.9 mg/100 g FM) and procyanidin B2 (16.8 mg/100 g FM) of all flavonols [47]. Du et al. [35] analysed the phenolic compounds contents in the fruit of different *Chaenomeles species*. The highest content of chlorogenic acid was noted for the following species: *C. speciosa* (1.82 mg/g FM), *C. thibetica* (1.17 mg/g FM) and *C. cathayensis* (1.19 mg/g FM), while in the fruit of *C. japonica* and *C. sinensis*, chlorogenic acid was found at low levels of 0.10 mg/g FM and 0.09 mg/g FM, respectively. The catechin content was 1.56 mg/g FM, 1.13 mg/g FM and 0.54 mg/g FM, while the procyanidin B1 content was noted at the levels of 2.22 mg/g FM, 1.45 mg/g FM, and 0.83 mg/g FM for the species *C. thibetica*, *C. cathensis* and *C. speciosa*, respectively. The species *C. speciosa*, *C. sinensis* and *C. japonica* were characterised by a high content of epicatechin (2.35 mg/g FM, 0.54 mg/g FM and 1.02 mg/g FM, respectively) and procyanidin B2 (2.96 mg/g FM, 0.40 mg/g FM and 0.98 mg/g FM, respectively). Strugała et al. [9] noted higher amounts of phenolic compounds in the fruit of the *C. speciosa* species. Procyanidin B1 was found at a level of 3.60 mg/g DM extract, procyanidin B2 at a level of 115.88 mg/g DM extract and epicatechin at 123.31 mg/g DM extract. According to Lewandowska et al. [48], flavonols account for 53.5% of all polyphenols, while catechin and epicatechin for 16.0% and 9.8% of the total polyphenols content, respectively.

The vitamin C content in the fruit is determined by the plant genotype, the year of harvest, as well as the air humidity and temperature during the growing period. Depending on the environmental conditions, the average vitamin C content in the Japanese quince fruit ranged from 172.6 to 243 mg/100 g [49]. The research results show that the fruit harvested in a warmer and drier year is characterised by a higher vitamin C content. The vitamin C level in the fruit is also determined by the duration of its storage. After one week of storage of the fruit, vitamin C losses of 15.5% in 2009 and 6.3% in 2010 were noted. After a two-week storage period, higher losses of vitamin C were observed in the fruit, i.e., 24.3% in 2009 and 15.8% in 2010 [49]. Byczkiewicz et al. [50] showed the presence of other vitamins in the *Chaenomeles varieties* studied. Cido (*Chaenomeles japonica*) and Maksim (*Chaenomeles x californica*) varieties had the highest content of niacin (2.04 and 2.07 mg/100 g, respectively). On the other hand, the highest levels of vitamins A and E were observed in the Cido variety (171.40 µg/100 g and 1.50 mg/100 g, respectively). The Maksim (*Chaenomeles x californica*) variety was characterized by the highest content of vitamin C (113.16 mg/100 g). In addition, the authors demonstrated the presence of riboflavin, thiamine, pantothenic acid and pyridoxine in *Chaenomeles varieties*. *Chaenomeles* is also a valuable fruit in terms of its content of minerals, including magnesium, calcium, potassium, sodium, zinc, iron and copper. Potassium, in particular, deserves special attention, as it has the largest proportion of all the above-mentioned components. Its content in the species *C. speciosa* is 84.7–147.0 mg/100 g, while in *C. japonica*, it is 249.0 mg/100 g [33].

Using gas chromatography, 21 volatile aromatic compounds were identified in the Japanese quince, including esters, aldehydes, alcohols, ketones and terpenes. In *C. japonica*, the proportion of alcohols was 39–73%, esters 6-18% and ketones 6–53%. Terpenes accounted for 5.2% of all volatile compounds found in this fruit. These components have a decisive influence on the taste qualities and characteristic aroma of the fruit [9,44]. Watychowicz et al. [33] confirmed that *C. sinensis* contained five groups of aromatic compounds: alcohols (hexanol, diacetone alcohol, (Z)-3-hexanol, (E)-2-hexanol, octanol, β-dihydroionol, propanol, 2-methyl propanol, butanol, 1-penten-3-ol, 3-methylbutanol, pentanol, cyclopentanol, benzyl alcohol, 2-phenyl ethyl alcohol); aldehydes (hexanal, heptanal, (E)-2-hexenal, (E)-2-heptanol, nonanal, (E,E)-2,4-decadienal, benzaldehyde); ketones (methyl isobutyl ketone, α-lonone, β-ionone, acetone, diisopropyl ketone, carvone); esters (isobutyl octanoate, butyl 7-octanoate, isobutyl 7-octanoate, hexyl hexanoate, ethyl hexanoate, hexyl ethanoate, ethyl 5-hexenoate, ethyl (Z)-4-hexanoate, (Z)-3-hexenyl acetate, 5-heksyl acetate, (E)-2-hexyl acetate, hexyl isobutyrate, isobutyl hexanoate, (Z)-3-hexenyl isobutyrate); and terpenes (limonene).

## 4. *Chaenomeles* Fruit-Based Products

*Chaenomeles* fruit is hard, astringent and acidic and therefore not intended for direct consumption. Nevertheless, it provides good material for the production of juices, syrups, candied fruit, jams, jellies, wines and liqueurs [7,8]. The literature also presents studies concerning the bioactive compounds and fatty acids found in the seeds, oil extracted from the seeds and gum produced from the seeds [4,8,51,52,53,54,55]. Only a few reports describe bioactive compounds in jams and candied *Chaenomeles* fruit. There is a lack of information on both the production technology and the physicochemical characteristics of fruit wines and liqueurs.

The presence of selected compounds in processed *Chaenomeles* sp. fruits is shown in Table 2.

### 4.1. Freeze-Dried Fruits

Antoniewska et al. [57] noted that freeze-dried Japanese quince contains numerous bioactive compounds. The vitamin C level was especially high and amounted to 287 mg/100 g. In addition, the total content of polyphenols was 4165 mg GAE/100 g and the antioxidant activity, determined on the basis of radical scavenging activity (DPPH), was 69%. The authors showed the presence of three groups of phenolic compounds in these products: phenolic acids with the dominant protocatechuic acid (1364 mg/100 g); flavan-3-ols, with the dominant ones being epicatechin and epigallocatechin gallate (364.1 mg/100 g and 246.6 mg/100 g, respectively); and flavonols, such as kaempferol and isoquercetin (66.52 and 5.27 mg/100 g, respectively). The research also identified the presence of 35 volatile compounds: alcohols (6), aldehydes (10), ketones (5), terpenes (10), furan compounds (3), carboxylic acids (3) and other compounds (3) [57].

### 4.2. Juice

According to Ros et al. [34], the juice fractions in the fruit of the species *C. japonica*, *C. x superba*, *C. speciosa* and *C. cathayensis* account for 42–52%, 51%, 46% and 44% of the fruit, respectively. The density of juice was found to be in the range from 1.020 to 1.039 g/mL, viscosity ranged from 1.06 to 1.32 cP and the pH value was 2.5–2.9. The low pH value is due to the high acidity which, for the above-mentioned species, is on average 3.5%, expressed as citric acid. Hellín et al. [41] identified three organic acids in juice, i.e., malic, quinic and succinic acid. That study reported that the proportion of malic acid was the highest, with its content ranging from 3.06 g/100 mL in a juice from the fruit of *C. japonica x C*. *speciosa* to 5.09 g/100 mL in a juice from the fruit of *C. cathayensis*. The quinic acid content ranged from 0.62 to 2.27 g/100 mL of juice, and the highest content of this compound was noted for *C. speciosa*. Succinic acid was found at the lowest levels of 8.20–174 mg/100 mL of juice, and its highest content was noted for *C. speciosa*. In another study, Hellin et al. [56] confirmed the presence of three organic acids in genotypes of *C. japonica*, namely, maleic acid (2.27–4.84 mg/100 mL), quinic acid (0.50–2.50 mg/100 mL) and succinic acid (0.004–0.012 mg/100 mL). Nine carbohydrates have been identified in the Japanese quince juice. The main carbohydrates determined in juice from different *Chaenomeles* species included monosaccharides, namely, fructose (0.73–2.29 g/100 mL) and glucose (0.31–1.07 g/100 mL), with the remaining sugars being sucrose (0.01–0.10 g/100 mL) and xylose (0.045–0.212 g/100 mL). The greatest amounts of fructose and glucose were observed in *C. cathayensis*, while the greatest amounts of sucrose were found in *C. japonica* [41].

*Chaenomeles* fruit juice is characterised by a high total phenolic compounds content, which is 185–413 mg/100 mL for *C. japonica*, 476 mg/100 mL for *C. speciosa*, 428 mg/100 mL for *C. cathayensis* and 421 mg/100 mL for *C. x superba* [34]. The main phenolic acids, which are present in free and bound form in Japanese quince juice, are mainly chlorogenic acid (26.9 mg/kg), caffeic acid (24.2 mg/kg) and coumaric acid (22.8 mg/kg), while the main flavonoids are epicatechin (148.4 mg/kg), catechin (55.5 mg/kg) and quercetin (12.3 mg/kg) [50]. The vitamin C concentration in fruit juice is high as well. Ros et al. [34] reported that in *C. x superba*, the content of this compound amounted to 75 mg/100 mL of juice, while *C. cathayensis* contained 61 mg/100 mL of juice, *C. japonica* contained 20–112 mg/100 mL of juice and *C. speciosa* contained 58 mg/100 mL of juice [34].

Regarding volatile compounds, Jordán et al. [9] reported the differentiated profiles of alcohols, aldehydes, ketones, esters and terpenic hydrocarbons in *Chaenomeles* sp. juice; for example, the dominant alcohols in juice from *C. japonica x C. speciosa* fruit were methanol and ethanol, while ethanol and 1-penten-3-ol were found in the greatest amounts in juice from *C. japonica*. The authors highlighted the role of volatile compounds in forming the flavour and fragrance of particular *Chaenomeles* species.

### 4.3. Sugar Syrup

Sugar syrup, i.e., juice extracted with sucrose, prepared on the basis of the Japanese quince fruit is also a good source of bioactive compounds [49,58]. Nevertheless, it was observed that the process of chopping the fruit and obtaining sugar syrup resulted in a reduction in vitamin C level by approx. 60% compared to the fresh fruit. The level of this compound was significantly affected by the pasteurisation process and long-term storage of syrup. Bieniasz et al. [49] noted that the vitamin C losses due to the pasteurisation process were approx. 7.5%, while a 15-week storage period of pasteurised sugar syrup resulted in losses ranging on average from 45% to 75%. According to Rubinskiene et al. [58], the vitamin C contents in sugar syrups prepared from the *Chaenomeles japonica* cultivar of Lichtar and a hybrid clone C.47 were 35.6 and 56.4 mg/100 g DM, respectively, and the total polyphenols contents were noted at the levels 265.9 and 100.08 mg/100 g DM, respectively. The antioxidant activity, determined as the DPPH radical scavenging ability of syrup, was 9.95 and 7.12 µmol TE/g, respectively. The syrups were characterised by low titratable acidities of 3.18 and 3.09%, respectively [58].

### 4.4. Candied Fruit

It was reported that Japanese quince fruit can be used for the production of candied fruit [3,58,59]. Seglina et al. [3] observed that the production method had a significant effect on the colour, concentration of phenolic compounds and vitamin C content in candied fruit. In a study by Rubinskienė et al. [58], candied fruits were characterised by a higher colour brightness than fresh fruit. The fruit that was candied and then convectionally dried using forced air circulation was darker in colour than fruit that was candied and microwave vacuum-dried [3]. It was found in the study that statistically significant differences occurred in terms of lightness L* and yellowness b* between the fruit dried with microwaves and by the convective drying method [3].

Krasnova et al. [59] noted that Japanese quince fruit candied in 50% sugar syrup by blanching it at 80 °C for 10 min had a comparable vitamin C content and higher phenolic compounds concentration than the fruit candied by keeping fruit pieces in sugar syrup at 60 °C for 10 min. It was also observed that the storage time had an effect on the phenolic compounds and vitamin C levels in candied fruit. After 120 days of storage, the concentration of phenolic compounds decreased by around 17%, while the vitamin C concentration decreased by around 40% in products candied and then dried using the convective or microwave method [3]. In the experiment conducted by Rubinskienė et al. [58], the vitamin C content was 116 and 124 mg/100 g DM, while the phenolic compounds content was 762 and 917 mg/100 g DM, depending on the fruit cultivar used for candying.

### 4.5. Jam

The literature that describes studies concerning a *Chaenomeles* fruit jam with no addition of other fruit is scarce. Only a study by Komar-Tyomnaya and Dunaevskaya [60] compared Japanese quince jams with industrially produced jams from apricot and apple, in terms of the content of essential elements. The authors demonstrated that the potassium content in jam from *Chaenomeles* fruit was high and amounted to 2087.2 mg/100 g, which seven times covers daily requirement of the human organism. As reported by Komar-Tyomnaya and Dunaevskaya [60], *Chaenomeles* jam was also rich in calcium and magnesium. It was characterised by calcium and magnesium contents 1.4 times higher than in apple jam, and the calcium content was 3.3 times than higher than in apricot jam. In addition, that jam was demonstrated to contain iron, zinc, copper and manganese as well [60].

Japanese quince fruit can be used as an addition to other fruit jams. An increase in the epicatechin content with an 8% addition of the Japanese quince fruit to gooseberry and strawberry jams was observed. The vitamin C concentration in gooseberry jam was rather low and amounted to 12.8 mg/100 g. The enrichment of gooseberry jams with an 8% addition of the Japanese quince fruit increased the vitamin C level by 50% [61]. The enrichment of gooseberry and strawberry jams with the Japanese quince fruit contributed to a decrease in the anthocyanin levels, while an increase in the total flavonoids and polyphenols levels, as well as DPPH radical scavenging activity, was observed [61,62]. Rumpunen and Göransson [68] conducted a sensory evaluation of jam from the Japanese quince fruit, using a hedonic scale of 1–9 points. According to consumers’ evaluation, the general appearance and the acidity of the product were highly rated at 7.4 and 6.5, respectively, while the overall impression of the jam was given a score of 5.8.

### 4.6. Powder

Powdered food is gaining popularity these days, both as an additive and as an end product. One of the techniques for obtaining powders is lyophilization, which extends the shelf life of the product. For example, powdered fruit can be used in the confectionery and bakery industry as an addition to teas, puddings and sauces [63]. The literature describing the research on the addition of powdered Japanese quince fruit to cookies is scarce, and the only studies related to this subject were conducted by Antoniewska et al. [57] and Turkiewicz et al. [64]. According to the research, the enrichment of cookies with 0.5% Japanese quince powder resulted in a two-fold increase in the antioxidant activity compared to the control sample, and the higher the amount of powder added to cookies, the higher the antioxidant activity. The storage of the cookies (16 weeks) significantly decreased the antioxidant activity of products. The authors also reported the impact of fruit powder on the oxidative stability of fat extracted from cookies, expressed as peroxide and anisidine values. Peroxide value is a measure of primary oxidation products, and the anisidine value reflects the presence of secondary oxidation products. It was found that higher amounts of Japanese quince fruit powder added to cookies (3–9%) resulted in higher peroxide and anisidine values, while powder added at 0.5–1.5% reduced both indices of oxidative changes compared to the control sample. In addition, after 12 weeks of storage, the degradation of hydroperoxides in the fat in cookies caused a decrease in hydroperoxide value, and an increase in anisidine value was simultaneously noted. The authors explained that these changes were due to the oxidation of unsaturated fatty acids in the lipid fraction of cookies [57].

Turkiewicz et al. [64] investigated Japanese quince fruit juice powders obtained with the use of carriers such as maltodextrin, inulin and a mixture of inulin with maltodextrin to facilitate the process of drying and using various drying techniques (vacuum, freezing and spraying). The authors showed that the drying method had a significant impact on the content of phenolic compounds. The highest content of total phenolics was found in powders manufactured using the freeze-drying method, while the lowest was found in samples prepared by vacuum drying. Moreover, differences between the powders with different carriers used were shown. The samples with maltodextrin had the highest content of total phenolic compounds, and the lowest value was observed for the powder with a mixture of maltodextrin and inulin. Powders prepared with the addition of maltodextrin were characterized by the highest content of phenolic acids and flavan-3-ols, while the highest level of polymeric procyanidins was noted in sample with inulin.

### 4.7. Chips

The latest data point to the search for new products from the Japanese quince fruit. Kowalska et al. [65] investigated the water activity, texture, acoustic properties, colour parameters and sensory properties of chips made from Japanese quince fruit with osmotic solutions (sucrose solution, juice concentrates of chokeberry and apple and mixture of chokeberry concentrate with sucrose solution) and then dried using convection, hybrid convection–microwave–vacuum and freeze-drying. The research showed that the type of osmotic solution and the drying technique had a significant effect on the mechanical and acoustic properties as well as the colour of the dried material. Sucrose syrup and apple juice concentrate used as osmotic solutions caused the smallest colour changes. The convection drying method resulted in the browning of the chips, and the freeze-drying method resulted in a smaller colour difference compared to the raw material [65].

### 4.8. Seeds

Japanese quince seeds are usually discarded in the fruit processing industry, which poses a major problem due to the high cost of waste disposal. In view of the possibility of reducing costs and unlocking a potentially good source of bioactive compounds, two advantages could be achieved: namely, the seeds can be used to create new products, and a waste-free fruit processing technology can be applied. The study results showed a high percentage of seeds in fruit waste after the application of various methods for fruit processing (29.8–38.3%) [8]. The presence of phenolic acids was demonstrated in Japanese quince seeds. Protocatechuic acid was the predominant compound. In addition, chlorogenic acid, caffeic acid, vanillic acid, p-coumaric acid, syringic acid and gallic acid were shown to be present [69], which may encourage the use of the seeds, e.g., in ground form as an additive to other food products.

The seeds can be used for the production of oil. The seeds of *C. japonica* contain 6.1–10.1% oil in DM. The average moisture content of the seeds is 43%. The seeds of *C. japonica x C.* speciosa are characterised by a higher oil content of 16.8% DM, and the moisture content of these seeds is 40.5% [51]. Sipeniece et al. [70] stated that the harvest year had a significant effect on the oil content of the *Chaenomeles japonica* seeds. For example, in 2017, the presence of oil in seeds was found at the level of 11.60%, while in 2018, it was found at a level of 12.50%. In the study by Urbanavičiūte et al. [8], the yield of cold-pressed oil from Japanese quince seeds ranged from 4.9% to 7.1%, depending on the fruit-processing method. Oil extraction using the Soxhlet method enabled a higher oil yield of 14.6–17.3%. The highest yield of oil from Japanese quince seeds, obtained by both the cold-pressing method and by the Soxhlet extraction, was noted following the production of puree from the fruit, and the lowest yield was noted for the oil from the seeds remaining after pressing juice from the fruit [8]. Likewise, Górnaś et al. [54] reported that the yield of oil from the Japanese quince seeds varies and is affected by the extraction method. A higher yield was obtained using the Soxhlet extraction method (11.5%) and ultrasound-assisted extraction with hexane (11.9%) than that obtained by the cold pressing method (8.7%).

### 4.9. Oil

Oil from *C. japonica* seeds is characterised by a high iodine value (98.1) and a low acid value (2.4), while the oil of the species *C. japonica x C. speciosa* has a higher iodine value (108.4) and a lower acid value (1.6) [51].

Previous studies demonstrated that Japanese quince seed oil contained numerous bioactive compounds. Górnaś et al. [4] observed that the total phenolic compounds content in cold-pressed oil was 64.03 mg/kg of oil, and the antioxidant activity of the oil, determined on the basis of DPPH assay, was at a level of 84.49%. Japanese quince oil is a rich product in terms of sterol content. Its content ranged from 5416.2 mg/100 g in oil from the *C. japonica* cultivar Rasa to 6667.1 mg/100 g oil from the *C. japonica* cultivar Rondo [54].

Oil from Japanese quince seeds contains 13 fatty acids. Oleic acid C18:1 and linoleic acid C18:2 deserve special attention. The oil contains small amounts of acid belonging to the Ω-3 group, i.e., α-linolenic acid [4,51,52,53,54]. According to Górnaś et al. [54], the proportion of oleic acid in the seeds of *C. japonica* cultivars “Rondo”, “Darius” and “Rasa”, amounts to 33.4%, 34.6% and 35.4%, respectively. The proportion of linoleic acid in these cultivars is at the levels of 53.4%, 52.8% and 51.7%, respectively. [54]. Oil extracted from the seeds of *C. japonica x C. speciosa* is characterised by a higher proportion of the C18:1 acid (44.16%) and a lower proportion of the C18:2 acid (43.94%) [51]. Sipeniece et al. [70] showed that the harvest year and cultivar had a significant influence on fatty acids. Nevertheless, the authors noted that C18:2 had the highest share and C14:0 had the lowest share of investigated fatty acids. A high content of C18:1 acid in each year of the study was also observed. Moreover, the cultivar “Rasa” was characterized by a significantly higher level of C18:1 and lower level of C18:2 compared to the cultivars “Rondo” and “Darius”.

Japanese quince seed oil contains tocopherols, mainly α-tocopherol, β-tocopherol and γ-tocopherol. The tocopherol profile is dominated by α-tocopherol, which accounts for 97% of all tocopherols. β-tocopherol and γ-tocopherol are found at low and similar levels and account for 1.3% and 1.6% of tocopherols, respectively. The oil extraction method had no significant effect on the tocopherol content in the oil [54].

The colour of oil from Japanese quince seeds ranges from light-yellow to dark-yellow. Urbanavičiūte et al. [8] conducted an experiment concerning the oil colour depending on the fruit processing method and the oil extraction method. Statistically significant differences were noted between the L*, a* and b* values of oils, and they were more pronounced for the cold-pressed oils than for the oils obtained by the Soxhlet extraction method, while in terms of the refraction index, the differences were not significant. Amongst the oils manufactured by the cold-pressing method, oils obtained after puree production showed the highest L*, a* and b* values. Within oils obtained by the Soxhlet method, the one extracted from seeds after syrup manufacturing differed significantly from those obtained after juice and puree production. Its L* and b* values were lower, and its a* value was higher than the values noted for other oils.

### 4.10. Gum

Quite recently, the interest of researchers was directed towards hydrocolloids which are present in *Chaenomeles* seeds, and after removing the oil from seeds, these hydrocolloids usually constitute a by-product. Wang et al. [66] investigated the structure and properties of gum extracted from the seeds of *Chaenomeles sinensis*, considering it as a potential hydrocolloid, which could be used as an emulsifier, preservative or stabilizer in various branches of industry. The authors reported that the main polysaccharide fraction of the investigated gum was composed of glucose, arabinose, xylose, galacturonic acid and glucuronic acid in a form of a highly branched heteroxylan comprised of an (a→4)-β-D-Xylp-(1→2,4)-β-D-Xylp backbone with side chains 1,2-α-D-GlcpA, 1,2,3,5-L-Araf or 1,4- β-D-Glcp. The gum was characterised by its high viscosity and thermal stability and therefore could be used as a thickener and stabilizer in the production of food which requires sterilization [66,67].

## 5. Conclusions

As can be seen from the above review, all *Chaenomeles* sp. fruits show interesting physicochemical properties, however, with different potential for use in fulfilling human needs. It seems that *C. speciosa*, *C. thibethica*, *C. cathayensis* and *C. sinensis* are of interest for pharmaceutical and cosmetic industries. The attractive aroma of Japanese quince fruit, as well as the high content of phenolic compounds, vitamin C, organic acids and dietary fibre, are what give Japanese quince fruit its great potential as a raw material in the food industry. The fruit can be widely used in food processing, as it provides a good material for the production of fruit juices, jams, jellies, candied fruit, chips and powder. It is worth searching for innovative technologies of *Chaenomeles* processing. The seeds of this fruit can be used both in their ground form as an addition to meals and for the production of oil and gum. It is also worth seeking new possibilities for using *Chaenomeles* in gastronomy as a substitute for lemon juice or vinegar, which may enhance both the health-promoting properties and the sensory traits of foods. There are only a few reports on the physicochemical composition of *Chaenomeles* fruit jams, and the scientific literature lacks information on the production technology and physicochemical characteristics of wines and liqueurs from *Chaenomeles* fruit.

## Figures and Tables

**Table 1:** The content of selected compounds in *Chaenomeles* sp. fruits.

Species	Components	Reference
*C. speciosa*	Phenolic compounds: procyanidin B1: 3.60 mg/g DM chlorogenic acid: 1.82 mg/g FM epicatechin: 2.35 mg/g FM procyanidin B2: 2.96 mg/g FM Minerals: Potassium: 115.85 mg/100 g	[9] [35] [33]
*C. thibetica*	Phenolic compounds: chlorogenic acid: 1.17 mg/g FM	[35]
*C. cathayensis*	Phenolic compounds: chlorogenic acid: 1.19 mg/g FM	[35]
*C. japonica*	Minerals: Potassium: 249 mg/100 g Phenolic compounds: chlorogenic acid: 0.10 mg/g FM epicatechin: 1.02 mg/g FM procyanidin B2: 0.98 mg/g FM Aromatic compounds: alcohols, esters, ketones, terpenes	[33] [35] [9,44]
*C. sinensis*	Aromatic compounds: alcohols, aldehydes, ketones, esters, terpenes Phenolic compounds: chlorogenic acid: 0.09 mg/g FM epicatechin: 0.54 mg/g FM procyanidin B2: 0.40 mg/g FM	[33] [35]

**Table 2:** The presence of selected compounds in processed *Chaenomeles* sp. fruits.

Material	Components	Reference
Juice fruits	Alcohols: methanol, ethanol, 1-penten-3-ol Phenolic acids: gallic, caffeic, coumaric Flavonoids: epicatechin, catechin, quercetin Vitamin C Carbohydrates: glucose, fructose, sucrose, xylose Organic acids: malic, quinic, succinic Polyphenols	[9] [9] [34,56] [34] [41] [41,56] [56]
Freeze-dried fruits	Vitamin C, Carotenoids, Chlorophyll, Antioxidant activity Phenolic acids: protocatechuic acid, neochlorogenic acid, chlorogenic acid, caffeic acid Flavan-3-ols: epicatechin, epigallocatechin gallate, epigallocatechin, Procyanidin A2 Flavonols: isoquercetin, kaempferol	[57]
Candied fruits	Vitamin C Colour Polyphenols	[3] [3,58] [58,59]
Jam	Minerals: potassium, calcium, magnesium, iron, zinc, manganese Flavonoid: epicatechin Vitamin C, Anthocyanins, Polyphenols	[60,61,62]
Powder	Polyphenols: phenolic acid, flavan-3-ol, polymeric procyanidin.	[55,63,64]
Chips	Water activity Texture Acoustic properties Colour parameters Sensory evaluation	[65]
Gum	Glucose, arabinose, xylose, galacturonic acid, glucuronic acid, uronic acid	[66,67]

## Data Availability

Not applicable.
